# Which Behavioral, Emotional and School Problems in Middle-Childhood Predict Early Sexual Behavior?

**DOI:** 10.1007/s10964-013-9973-x

**Published:** 2013-07-04

**Authors:** Alison Parkes, Andrea Waylen, Kapil Sayal, Jon Heron, Marion Henderson, Daniel Wight, John Macleod

**Affiliations:** 1 CSO/Medical Research Council Social and Public Health Sciences Unit, University of Glasgow, 4 Lilybank Gardens, Glasgow, G12 8RZ UK; 2 School of Oral and Dental Sciences, University of Bristol, Bristol, UK; 3 Developmental Psychiatry, University of Nottingham, Nottingham, UK; 4 School of Social and Community Medicine, Centre for Academic Primary Care, University of Bristol, Bristol, UK

**Keywords:** Sexual behavior, Adolescent, Conduct problems, School engagement, ALSPAC

## Abstract

Mental health and school adjustment problems are thought to distinguish early sexual behavior from normative timing (16–18 years), but little is known about how early sexual behavior originates from these problems in middle-childhood. Existing studies do not allow for co-occurring problems, differences in onset and persistence, and there is no information on middle-childhood school adjustment in relationship to early sexual activity. This study examined associations between several middle-childhood problems and early sexual behavior, using a subsample (N = 4,739, 53 % female, 98 % white, mean age 15 years 6 months) from a birth cohort study, the Avon Longitudinal Study of Parents and Children. Adolescents provided information at age 15 on early sexual behavior (oral sex and/or intercourse) and sexual risk-taking, and at age 13 on prior risk involvement (sexual behavior, antisocial behavior and substance use). Information on hyperactivity/inattention, conduct problems, depressive symptoms, peer relationship problems, school dislike and school performance was collected in middle-childhood at Time 1 (6–8 years) and Time 2 (10–11 years). In agreement with previous research, conduct problems predicted early sexual behavior, although this was found only for persistent early problems. In addition, Time 2 school dislike predicted early sexual behavior, while peer relationship problems were protective. Persistent early school dislike further characterized higher-risk groups (early sexual behavior preceded by age 13 risk, or accompanied by higher sexual risk-taking). The study establishes middle-childhood school dislike as a novel risk factor for early sexual behavior and higher-risk groups, and the importance of persistent conduct problems. Implications for the identification of children at risk and targeted intervention are discussed, as well as suggestions for further research.

**Electronic supplementary material:**

The online version of this article (doi:10.1007/s10964-013-9973-x) contains supplementary material, which is available to authorized users.

## Introduction

Increasingly, public health researchers have acknowledged the development of sexuality in adolescence as a normal process, to be treated as one of the main “developmental tasks” of the teenage years (Tolman and McClelland 2011). Nevertheless, early sexual activity is likely to expose young people to unwanted sex (Martinez et al. 2011), sexually transmitted infections (Kaestle et al. 2005), and teenage pregnancy (Lau et al. 2013). There is therefore considerable interest in identifying predictors of early sexual behavior (sex before age 16), rather than the normative timing of 16–18 years for men (Martinez et al. 2006) and women (Chandra et al. 2005) in contemporary Europe and North America. Developmental theories such as problem behavior theory (Jessor and Jessor 1975) suggest that early timing of sexual behavior is allied with the emergence of other adolescent risk behaviors, pointing to pathways to early sexual activity that are likely to differ from those found for normative sexual development. A major review of longitudinal US research on adolescent sexual debut (Zimmer-Gembeck and Helfand 2008) found that biological and social factors, including earlier puberty, dating, family characteristics and family processes such as lower parental monitoring, distinguish teenagers who initiate sex before age 18 from those whose first experience is delayed. However, these factors did not appear to distinguish early sexual initiation (“early sex”) from normative initiation at 16–18 years. The review suggested that behavioral, emotional and school adjustment problems may represent a unique set of factors distinguishing early sexual behavior from normative timing.

Research tracing associations with early sexual behavior back to behavioral, emotional and school adjustment problems in middle-childhood (6–12) years may be particularly valuable in the early identification and targeting of populations at risk of poor sexual health. However, the few existing longitudinal studies of middle-childhood problems in relationship to the early timing of sexual behavior (Dupéré et al. 2008; McLeod and Knight 2010; Ramrakha et al. 2007; Schofield et al. 2008b) are limited in scope. In particular, they focus almost exclusively on externalizing problems. This neglects the role of depression, peer rejection and school maladjustment suggested by some theories of adolescent risk development. Existing research also neglects the importance of the onset and persistence of each problem type for early sexual behavior, and does not adequately allow for potential confounders. In what follows, we first provide information on the likely role of different problem types for early sexual behavior, the need to consider timing and persistence of each problem, and important confounders. As the timing of first sex is only one indicator of exposure to risk for sexually active adolescents, we then move to a person-centered approach to distinguish higher- and lower-risk subgroups among those reporting early sexual behavior. We consider research suggesting that middle-childhood problems may differentiate sexually active adolescents who are most at risk.

### Externalizing Problems

Within the broad category of externalizing problems, two main groups of problems have been linked with early sexual behavior: conduct problems, and problems with attention and/or hyperactivity (“attentional problems”). Theoretical grounds for linking conduct problems with early sexual behavior appear more strongly articulated in the literature than theories for attentional problems. One developmental perspective assigns an exclusive role to early conduct problems involving fighting and rule breaking. These behavioral problems are thought to show “heterotypic continuity”, first with delinquency and substance abuse in the early teens, and then with early sexual behavior (Moffitt 1993). In other words, both conduct problems and early sexual activity are simply different behavioral manifestations of the same underlying characteristics. A second perspective, that of interactional (Thornberry 1987) and transactional models (Dodge and Pettit 2003), argues that childhood conduct problems destabilize ties with the protective institutional framework of family and school embodied in problem behavior (Jessor and Jessor 1975) and social control (Hirschi 1969) models of adolescent risk development. Here, the child who is aggressive and difficult to manage becomes progressively alienated from family, school and “healthy” peers, and closer to delinquent peers. For attentional problems, analogous models could apply. Underlying temperamental deficits such as low effortful and reactive control (Martel and Nigg 2006) might lead adolescents with attentional problems to engage in health risk behavior. Attentional problems also have been associated with reduced school engagement (Kofler et al. 2008) and poorer academic performance (Barbaresi et al. 2007), and the disruptive behavior characteristic of such problems may prove unacceptable to peers (Barkley et al. 2006). In this way, attentional problems might trigger reduced ties with school and “healthy” peers, and encourage the child to adopt less conventional norms and friendships.

Existing longitudinal studies examining associations between middle-childhood problems and early sexual behavior (Dupéré et al. 2008; McLeod and Knight 2010; Ramrakha et al. 2007; Schofield et al. 2008b) or sexual risk behavior (Fergusson et al. 2005; Fontaine et al. 2008; Wu et al. 2010; Timmermans et al. 2008) have all found associations for middle-childhood conduct problems, aggressive or antisocial behavior. While comorbidity of conduct and attentional problems is common in middle-childhood, there is conflicting information on the role of attentional problems. Some research found no effect of concurrent middle-childhood hyperactivity/inattention on early sexual behavior (Ramrakha et al. 2007) or other sexual risk outcomes including teenage pregnancy (Fergusson et al. 2005); while two other studies suggested independent contributory effects of attentional problems on early sexual behavior (McLeod and Knight 2010; Schofield et al. 2008b) and a third on early pregnancy (Fontaine et al. 2008). Although there are known gender differences in the prevalence of both middle-childhood externalizing problems and early sexual behavior, none of the above-mentioned studies has found gender differences in the effect of conduct or attentional problems on early sexual behavior or sexual risk-taking.

### Other Middle Childhood Problems: Internalizing Problems, School Maladjustment, and Peer Relationship Problems

In addition to the role of externalizing problems, there are grounds for supposing that other middle-childhood problems also may be linked to early sexual behavior. Within the broad group of internalizing problems, there may be different effects of anxiety and depression. Prospective associations between anxiety and some other forms of adolescent risk behavior appear inconsistent (Zehe et al. 2013), and may depend partly on whether anxiety is generalized or social, and on the influence of peer norms and behavior. The effects of anxiety on risk behavior also may be mediated by concurrent depression (Frojd et al. 2011). Depression is associated with low self-esteem (Orth et al. 2008), interpersonal skills (Nilsen et al. 2013) and support from family and “healthy peers” (Auerbach et al. 2011); and all of these factors are likely to make a teenager with depression vulnerable in situations leading to risk behavior. Poor school engagement and performance are interrelated aspects of school maladjustment, which might be linked to early sexual behavior via underlying cognitive deficits leading to poor judgment (Halpern et al. 2000), or via depressed educational expectations and aspirations that may strengthen ties with delinquent peers (Crosnoe 2002). Peer relationship problems also might be important, although it is unclear whether they are harmful or protective. A child who is not accepted by classmates may substitute a lack of ties to “healthy” peers with ties to “delinquent” peers and adoption of unconventional norms, which in turn may fuel risk behavior (Deater-Deckard 2001). Alternatively, as sociability and popularity seem critical for sexual behavior (Meschke et al. 2000), a child who finds it difficult to make friends may be less likely to engage in early sexual behavior. Thus, according to this second model, peer relationship problems could be protective.

Within the group of internalizing problems, two studies have found protective or null effects of middle-childhood anxiety on early sexual timing and subsequent sexual risk (Ramrakha et al. 2007; Fergusson et al. 2005). A third study (McLeod and Knight 2010) produced a null finding for internalizing symptoms as a group, although this may have concealed a risk effect of depression and protective or null effects of anxiety. Early adolescent depression has been prospectively linked to early sexual behavior in girls but not boys (Whitbeck et al. 1999), and to sexual risk-taking in both sexes (Lehrer et al. 2006), but we lack information for the predictive power of depression in middle-childhood. A null effect has been found on early sexual behavior for middle-childhood peer problems (McLeod and Knight 2010).

Research on associations between middle-childhood school adjustment and early sexual behavior is lacking. We therefore turn to longitudinal studies of early adolescent school engagement and performance. Various aspects of school engagement including dislike of school (Bonell et al. 2005), reduced participation in school activities (Whitbeck et al. 1999) and lower educational aspirations (Smith 1997) have been found to predict early sexual behavior and/or sexual risk-taking. The first study examined females only, but the two remaining studies found effects stronger among females (Whitbeck et al. 1999) or confined to females (Smith 1997). Longitudinal research also found lower school grades predicted sexual initiation (Santelli et al. 2004), echoed in a second study that found no gender differences in the effect of school performance (Laflin et al. 2008).

### Timing and Persistence of Middle-Childhood Problems

The timing of problems may have implications for predictive power in relationship to early sexual behavior. The importance of early timing of conduct problems has been suggested by Moffitt’s taxonomy of conduct problems, dividing these into early life-course persistent or adolescent-limited (Moffitt 1993). Greater lifetime risk involvement is found for the former group (Moffitt et al. 2002). As already described, much previous research on the development of adolescent risk behavior has given central place to externalizing problems (particularly conduct problems), which are the most common mental disorder in young children. An alternative perspective allows for the possibility that other types of middle-childhood problem timed well before puberty might be predictive of early sexual behavior. In developmental “cascade” models (Masten and Cicchetti 2010), the main focus is on how one type of problem may “spill over” into another type over the course of a child’s development. For our purposes, however, the interest lies in the origins, rather than the consequences, of cascades: the cascade process may originate with early academic failure, internalizing or externalizing problems in middle-childhood and transition to any of the others (Moilanen et al. 2010). These multiple possible origins suggest a greater profusion of possible pathways to early sexual behavior from various types of early problem originating well before puberty, involving depression and school maladjustment as well as externalizing problems.

Despite the likely importance of early timing, some middle childhood problems may not surface until puberty. For instance, while externalizing problems are the most common mental disorder in young children, internalizing problems become more common around puberty (Costello et al. 2003). Puberty is also likely to be a particularly vulnerable transition period in terms of physical maturation, greater independence from parents and increased peer influence. Recent work on cascading problems also supports the view of a vulnerable period, with a greater number of problem transitions around this time (Moilanen et al. 2010). Two key questions are first, whether some problems have more impact at puberty than earlier in development; and second, whether problems at puberty can add to the predictive power of other earlier problems for adolescent risk behavior. On the first point, it seems likely that some problems arising at puberty may have a greater impact on the child than similar difficulties arising earlier. Deficiencies in basic literacy and numeracy skills may become less easy to correct and have more pervasive effects after children move to secondary school, while older children may be more susceptible to the modeling of antisocial behavior by deviant peers (Dishion et al. 1999). In regards to the second question, as we have seen, early conduct problems may elicit later problems nearer puberty, including depression, peer rejection and school maladjustment. Later problems that lie on the same pathway as early conduct problems may not add to the predictive power of the earlier problems. Alternatively, later problems may add to risk; support for the idea of cumulative risk comes from a study that found pathways to teen antisocial behavior from both early externalizing problems and later peer rejection (Laird et al. 2001), but there is currently no information on whether both early and later middle-childhood problems impact on early sexual behavior.

In addition to the onset, the persistence of problems warrants consideration. Not all problems with an early onset persist throughout middle childhood; for example, less than half of the children with early conduct problems will show persistent problems (Barker and Maughan 2009), and not all children maintain a stable level of school engagement through middle childhood (Ladd and Dinella 2009). In young adulthood, research showed that increased health risks predicted by childhood conduct problems were confined to those with persistent early problems, while those with child-limited problems in general had similar risks to the cohort norm of low problems (Odgers et al. 2008). A longitudinal study of adolescents also demonstrated the importance of life-course persistent conduct problems during adolescence, which were associated with greater sexual risk-taking, compared to either late onset or adolescent-limited problems (Aalsma et al. 2010).There are, however, gaps in our knowledge of how differences in the onset and persistence of problems in middle-childhood may affect early sexual behavior. Two studies found that early and increasing levels of conduct problems (Wu et al. 2010) and physical aggression (Timmermans et al. 2008) over middle-childhood in both boys and girls were associated with higher levels of substance use combined with sexual risk taking, and the third study of females only (Fontaine et al. 2008) found that that early high hyperactivity combined with high physical aggression predicted early pregnancy. Although these studies suggest the importance of persistent early childhood conduct problems for higher-risk groups, they do not specifically assess early sexual behavior or consider a full range of middle-childhood problems.

### Potential Confounders of Associations Between Middle-Childhood Problems and Early Sexual Behavior

Several underlying influences may be responsible for observed associations between middle-childhood problems and adolescent risk behaviors, relating to family circumstances, family functioning and child characteristics. Family adversity is associated with children’s mental health (Essex et al. 2006) and school performance (Frederickson and Petrides 2008), and aspects of family circumstances indicative of greater adversity such as low parental occupational class and fathers’ absence from the household have been linked with adolescent risk behaviors including sexual behavior (Zimmer-Gembeck and Helfand 2008). Parenting practices are intertwined closely with the development of children’s behavioral problems (Stormshak et al. 2000) and school engagement (Simons-Morton and Chen 2009); and parent–child connectedness, parental knowledge and monitoring of behavior, and parental values supportive of delayed sex are all associated with a lower risk of early sexual behavior (Miller et al. 2001). Early pubertal timing is known to predict early sexual initiation (French and Dishion 2003) as well as being associated with other problem behavior (Halpern et al. 2007). Turning to other child characteristics, cognitive functioning has been implicated in several problems, including conduct disorder (Boden et al. 2010), and also linked with adolescent sexual behavior (Halpern et al. 2000). Physical attractiveness may be related negatively to both peer relationship (Vannatta et al. 2009) and internalizing problems (Rosen et al. 2011), while romantic affiliations may serve as an additional stimulus to antisocial behavior over and above the influence of parenting and delinquent friends (Lonardo et al. 2009). In turn, both physical attractiveness and romantic involvement are important for early sexual behavior (Zimmer-Gembeck et al. 2004). In order to minimize the possibility that shared influences are responsible for associations found between middle-childhood problems and early sexual behavior, it is important to take account of these factors. However, few existing studies examining associations between middle-childhood problems and early sexual behavior adjusted for potential confounders beyond measures of family adversity.

### Middle-Childhood Problems and Adolescent Sexual Risk Group Membership

As already noted, the development of sexuality in adolescence may proceed along different pathways leading to early, normative or later timing (Zimmer-Gembeck and Helfand 2008). Of course, the concept of what is early, normative or late is necessarily somewhat imprecise, varying with cultural and historical influences, and timing is only one indicator of adolescent sexual health risk (Hawes et al. 2010). It is likely that among adolescents experiencing early sexual behavior, there are some for whom sexual development closely resembles that of other adolescent risk behaviors, and others for whom the process is more normative. For the first group, the pathway to early sexual behavior may be via delinquent peer affiliation (Crockett et al. 2006) and consequent antisocial behavior and substance use in the early teens (Schofield et al. 2008a). Adolescents who take such a “high-risk” pathway to early sexual behavior, via other risk behaviors, are also likely to engage in greater sexual risk-taking (Siebenbruner et al. 2007). For the second group experiencing early sexual behavior, a more normative pathway might be dictated largely by earlier timing of biological and social influences such as puberty and romantic involvement, and we might expect these adolescents to take fewer sexual risks. Research on the developmental pathways associated with life-course-persistent and adolescent-limited conduct problems suggests a greater accumulation of early problems for the former group (Moffitt and Caspi 2001). As already noted, earlier onset and growth of conduct problems have been linked to higher adolescent risk involvement, including sexual risk-taking (Wu et al. 2010). However, little is currently known about whether onset and persistence of other middle-childhood problems differentiate higher-risk sexual development.

## Current Study and Hypotheses

The current study uses a nationally representative population cohort to investigate three main questions. The first two questions adopt a variable-centered approach, to ask which types of middle-childhood problem are predictive of early sexual behavior, and whether the timing and persistence of each problem during middle-childhood matters. We adopt a developmental perspective suggesting multiple pathways to early sexual behavior, from more than one type of problem. This allows for the possibility that, while they may be important from early on, some problems may be added to by other problems that become important at late primary school age. We examine the roles of hyperactivity/inattention, conduct problems, peer relationship problems, depressive symptoms, school attainment and school dislike, considering whether there are gender differences in the effect of any of these problems. (We do not consider anxiety, since earlier studies suggest null or protective effects associations with sexual behavior (Zimmer-Gembeck and Helfand 2008).) We examine problems at two time points (6–8 years, and 10–11 years). In order to minimize the possibility that shared influences are responsible for associations found between problems and early sexual behavior, we allow for important factors associated with the development of sexual behavior, which also are implicated in the development of middle-childhood problems, namely family adversity, parenting and child characteristics.

In relationship to the first research question, we hypothesize that conduct problems will be associated with early sexual behavior, in line with existing longitudinal research that has allowed for a more restricted range of confounding influences (Dupéré et al. 2008; McLeod and Knight 2010; Ramrakha et al. 2007; Schofield et al. 2008b). In relationship to the role of other behavioral and emotional problems (hyperactivity/inattention, depression and peer relationship problems), there is insufficient information on which to base a hypothesis. For attentional problems, two existing studies with null findings for early sexual behavior and sexual risk taking allowed for a much greater range of possible confounders than studies with positive findings. For peer relationship problems, the direction of any association with early sexual behavior is unclear: they might constitute risk or be protective if sociability is important for early sexual behavior, and the one existing study found no effect. There were no studies examining middle-childhood depression or school maladjustment in relation to early sexual behavior. Although existing research suggests no gender differences in the effect of conduct problems on early sexual behavior, studies of depression and school maladjustment in early adolescence suggested that effects were stronger in females. Accordingly, we test for gender differences in the effect of all middle-childhood problems.

In regards to the second question about timing and persistence, limited previous research suggests a central role for early life-course-persistent conduct problems in the development of adolescent risk, but little effect for child-limited or later onset conduct problems. While there is little known in relationship to sexual timing, we hypothesize that only persistent early conduct problems, rather than increasing or decreasing problems, will be associated with early sexual behavior. Again, there is insufficient information to form hypotheses for the other types of problem, although from research on the development of antisocial behavior we would expect some later-onset problems to add to any risk predicted by early conduct problems (Laird et al. 2001).

The third research question uses a person-centered approach to ask which types of middle-childhood problem are predictive of different risk subgroup membership within the group reporting early sexual behavior. We classify membership of risk subgroups in two alternative ways: first, according to engagement in other early adolescent risk behaviors as a precursor to early sexual behavior; and second, according to other sexual risk exposure beyond sexual initiation. We expect some middle-childhood problems to be predictive of both high- and low-risk subgroups reporting early sexual behavior. However, we also expect problems to be identified more strongly with the development of early sexual behavior in tandem with other adolescent risk behavior, compared with more normative pathways. Research indicates greater accumulation of childhood risk factors for higher-risk, life-course persistent compared with lower risk, adolescent-limited antisocial behavior groups (Moffitt and Caspi 2001). We therefore hypothesize that a greater number of persistent early middle-childhood problems will be associated with membership of a higher-risk subgroup among all adolescents reporting early sexual behavior.

## Method

### Participants

The Avon Longitudinal Study of Parents and Children (ALSPAC) (Boyd et al. 2012) aimed to recruit all pregnant women resident in the county of Avon in England, U.K. whose expected date of delivery lay between 1 April 1991 and 31 December 1992. 14,541 pregnant women were enrolled between these dates, representing about 85 % of the eligible population (Golding 2004). Mothers consented to join the study at recruitment and were free to withdraw at any time. Compared to 1991 National Census Data, the recruited sample was similar to the UK population, although with a slightly higher proportion of married or cohabiting mothers who owned their home and car, and a slightly smaller proportion from ethnic minorities. The study was approved by the ALSPAC Law and Ethics Committee and Local Research Ethics Committees.

### Data Set

The baseline “core” sample of children alive at 1 year was 13,988. Second-born twins, triplets and quadruplets were excluded from this study. Problems were measured using data gathered from mothers and children via postal questionnaires and clinic session interviews at two time points: Time 1, early primary school (6–8 years) and Time 2, late primary (10–11 years). Information on early teen risk behavior was gathered using postal questionnaires (age 13 years 7 months) and computer-assisted structured-interviews at a clinic session (average age 13 years 6 months). Information on covariates was gathered using postal questionnaires sent to mothers and children at intervals between the ages of 6 months and 15 years. Information on the main study outcome, sexual behavior, was gathered at the age 15 clinic (average age 15 years 6 months, N = 5,180) held between October 2006 and November 2008. Although all children were invited to attend clinic sessions, when compared with non-attendees, age 15 clinic attendees contained more children whose mother was married (81 vs. 71 %), educated to degree level (18 vs. 10 %) and from professional or managerial social classes (44 vs. 32 %). Ethnic composition was similar (98 vs. 97 % white). Further exclusions from the age 15 clinic sample were made of attendees who had not reached 15 years, did not start romantic relations questions or (since further questions related to behaviors with young people) said they did not spend any of their free time with other young people, leaving an eligible sample of N = 4,798.

### Measures

#### Early Sexual Behavior

Computer-assisted structured-interviews (CASI) used questions adapted from the Adolescent Sexual Activity Index (Hansen et al. 1999). Parents gave consent for children to take part, and teenagers assented to completing the CASI. The main outcome, early sexual behavior, was a positive response at the age 15 clinic session to either of two questions about oral sex and intercourse (“In the last year have you had oral sex with another teenager/young person? This is when they put their mouth or tongue on your penis/vagina or you put your mouth or tongue on their penis/vagina”; “In the last year have you had sexual intercourse with another teenager/young person?”). Both types of sexual behavior present health risks to adolescents, and a large US study found that around half of adolescents initiating any one form of sexual behavior (oral, vaginal or anal) reported other forms within a year (Halpern and Haydon 2012). Separate analyses using intercourse and oral sex gave similar results to the combined outcome used here.

An index of sexual risk-taking was created from a count of risks (range 0–5, with a cut-off of 3 or more denoting “high-risk sex”). Risks reported at the age 15 clinic comprised using alcohol and/or drugs at recent sex; condom not used at most recent sex, not always used; more than one partner. To these we added sex (oral and/or intercourse) reported at an earlier clinic (13½ years). Risk relating to intercourse was used to compile the sexual risk index, unless teenagers only reported oral sex: for these cases risk relating to oral sex was used.

#### Other Risk Behavior at 13 Years

“Age 13 risk” was measured from any substance use (cigarette smoked in last 6 months, ever tried cannabis, ever been drunk), or any involvement in serious antisocial behavior (car breaking, joy riding, carrying a weapon, damage to property, housebreaking, robbery with force or fire raising). Measures were selected on the basis of similar prevalence to prevent a composite risk measure from being dominated by any particular behavior.

#### Middle-Childhood Problems

This section describes measures, with sample information provided in Table 1. All problem measures were dichotomized to create binary indicators. The main advantage of this approach was for behavioral problems (hyperactivity/inattention, conduct problems and peer relationship problems), as this permitted assessment of problem levels indicative of psychiatric diagnoses. For other types of problem (depressive symptoms, school performance and school dislike) the cut-off points chosen were necessarily more arbitrary, and chosen for simplicity. Analyses using binary measures of problems were in all cases supplemented by analyses using continuous scores. Levels of problems in the eligible sample were below those in the full data set (all differences *p* < 0.001 with the exception of Time 1 depressive symptoms and school dislike, where differences were not statistically significant at the *p* < 0.05 level). Table 1 shows that levels of problems were generally higher in boys than girls, although there were no gender differences in depressive symptoms at Time 1, conduct problems or school performance at Time 2, and more depressive symptoms were found in girls than boys at Time 2.

**Table 1:** Measures used for middle-childhood problems from ALSPAC, and analysis sample information (N = 4,739)

Problem type and score range	Source of information, method of data collection and age of child	Total sample mean (SD)	Males mean (SD)	Females mean (SD)	Gender difference
Hyperactivity/inattention^a^ T1 (range 0–10)	Mother, postal questionnaire, 6 years	3.18 (2.28)	3.58 (2.37)	2.82 (2.13)	***
Hyperactivity/inattention^a^ T2 (range 0–10)	Mother, postal questionnaire, 11 years	2.59 (2.11)	2.97 (2.21)	2.25 (1.96)	***
Conduct problems^a^ T1 (range 0–10)	Mother, postal questionnaire, 6 years	1.51 (1.42)	1.57 (1.40)	1.46 (1.42)	*
Conduct problems^a^ T2 (range 0–10)	Mother, postal questionnaire, 11 years	1.12 (1.34)	1.12 (1.37)	1.12 (1.31)	
Peer relationship problems^a^ T1 (range 0–10)	Mother, postal questionnaire, 6 years	0.97 (1.32)	1.07 (1.39)	0.89 (1.25)	***
Peer relationship problems^a^ T2 (range 0–10)	Mother, postal questionnaire, 11 years	1.04 (1.46)	1.12 (1.49)	0.97 (1.42)	***
Depressive symptoms^b^ T1 (range 10–12)	Mother, postal questionnaire, 7 years	0.33 (1.01)	0.31 (1.00)	0.34 (1.02)	
Depressive symptoms^c^ T2 (range 0–26)	Mother, postal questionnaire, 11 years	2.23 (3.07)	2.12 (3.01)	2.32 (3.12)	*
School performance T1 (range 0–15)	Key stage 1 scores, linked data, 7 years (Year 2 of primary school)	10.58 (3.25)	10.25 (3.30)	10.88 (3.18)	***
School performance T2 (range 0–280)	Key stage 2 scores, linked data, 10 years (Year 6 of primary school)	194.83 (41.02)	194.20 (41.12)	195.38 (40.94)	
School dislike T1 (range 1–4)	Child, postal questionnaire and clinic session, both 8 years	1.92 (0.59)	2.01 (0.62)	1.84 (0.56)	***
School dislike T2 (range 1–4)	Child, postal questionnaire, 11 years	1.96 (0.59)	2.06 (0.64)	1.87 (0.54)	***

Higher scores indicate higher levels of problems, with the exception of school performance measures. *T* tests of gender differences: * *p* < 0.05, ** *p* < 0.01, *** *p* < 0.001

^a^Strengths and Difficulties Questionnaire (Goodman 1997, 2001)

^b^Development and well-being assessment (Goodman et al. 2000a)

^c^Short Moods and Feelings Questionnaire (Angold et al. 1995)

##### Behavioral Problems: Hyperactivity/Inattention, Conduct Problems and Peer Relationship Problems

These problems were reported by mothers using the Strengths and Difficulties Questionnaire (SDQ) (Goodman 1997), a widely-used instrument to measure children’s psychosocial adjustment, with high reliability and validity (Goodman 2001). Each of the three types of behavioral problem was measured using five items, asking the respondent to indicate, using a three-point scale, whether statements were “not true”, “somewhat true” or “certainly true”. Items measuring hyperactivity/inattention comprised “restless, overactive, cannot stay still for long”, “constantly fidgeting or squirming”, “easily distracted, concentration wanders”, “thinks things out before acting” (reversed), “sees tasks through to the end, good attention span” (reversed). Items measuring conduct problems comprised “often has temper tantrums or hot tempers”, “generally obedient, usually does what adults request” (reversed), “often fights with other children or bullies them”, “often lies or cheats”, “steals from home, school or elsewhere”. Items measuring peer relationship problems comprised “rather solitary, tends to play alone”, “has at least one good friend” (reversed), “generally liked by other children” (reversed), “picked on or bullied by other children” and “gets on better with adults than with other children”. Recommended cut-offs were applied for abnormal or borderline levels of problems (Goodman 1997), indicative of psychiatric diagnosis (Goodman et al. 2000b).

##### Depressive Symptoms

Maternal report of child depression was measured at Time 1 from the Development and Well-being Assessment (Goodman et al. 2000a) using the number of symptoms from a list of twelve items asked about if the child was said to be “very sad, miserable, unhappy or tearful” in the past month. These were: child lacked interest in past month, ate more/less than usual in past month, lost/gained a lot of weight in past month, found it hard to get to sleep in past month, slept too much in past month, frequently agitated for a period in past month, frequently felt worthless/guilty for a period in past month, found it unusually hard to concentrate in past month, thought about death a lot in past month, talked about harming/killing self in past month, tried to harm/kill self in past month, tried to harm/kill self in lifetime. At Time 2, depression was measured using the Short Moods and Feelings Questionnaire (Angold et al. 1995). This consisted of 13 items on the child’s behavior and feelings in the past 2 weeks: the child “felt miserable or unhappy”, “didn’t enjoy anything at all”, “felt so tired that he just sat around and did nothing”, “was very restless”, “felt he/she was no good any more”, “cried a lot”, “found it hard to think properly or concentrate”, “hated himself/herself”, “felt he/she was a bad person”, “felt lonely”, “thought nobody really loved him/her”, “thought he could never be as good as other kids”, “felt he/she did everything wrong”. In both instances the top 10 % of scores (any symptoms at Time 1, scoring 6 or more at Time 2) was used to indicate high levels of depressive symptoms.

##### School Problems: School Performance and School Dislike

Performance was measured via linkage to Key Stage 1 and 2 standardized National Curriculum assessments used in England^1^. Key Stage 1 of the National Curriculum covered years 1 and 2 of primary school (ages 5–7 years), and was assessed in year 2 (Time 1) using summary scores of teacher tests in reading, writing and mathematics (range 0–15 points). Key Stage 2 covered years 3–6 of primary school (ages 7–11 years) and was assessed in year 6 (Time 2), summary scores combining marks obtained in English, mathematics and science tests (range 0–280). At either time point, for simplicity, a “problem” with school attainment was defined as a score below the median. School dislike was self-reported by children. At Time 1, four items (Cronbach alpha = 0.69) asked how often they liked going to school, how often they felt happy at school, whether schoolwork was boring or interesting and how much they liked school. At Time 2, five items (Cronbach alpha = 0.85) asked for agreement with statements: “My school is a place where…I like to go each day, I like to be, learning is fun, I enjoy what I do in class, I get excited about the work we do”. Responses were coded so that high scores indicated more dislike. For simplicity, a “problem” with school dislike was defined as a score above the median value. Owing to tied observations, this gave unequal groups for school dislike: 29 % of the sample at Time 1 and 36 % at Time 2 were above the median score.

##### Covariates

These included child gender, age in months at the age 15 clinic session and stage of pubertal development assessed via a postal pictorial questionnaire issued at the same time as the clinic session. (Note that the sample ethnic composition was 98 % white, so ethnic group was not used as a covariate.) Additional covariates included key predictors of early sexual behavior identified in the literature, which also relate to middle-childhood problems. This section describes measures, with sample information provided in the Online Resource 1. In line with other research on early childhood problems and risk in young adulthood (Fergusson et al. 2005), we used multiple indicators to capture family adversity: maternal educational level and father’s social class (both measured at 32 weeks gestation), whether the mother had ever smoked (18 weeks gestation), whether the father was absent during any part of childhood (based on five measurements from birth to age 10), and household financial difficulties (based on questions about difficulty affording food, clothing, heating, rent/mortgage, or things for the child, and measured on four occasions during pregnancy and early childhood). Stage of pubertal development was measured using a pictorial questionnaire completed by the mother and/or child at ages 10 and 11. Parenting processes included parent–child connectedness and parental knowledge. Parent–child connectedness was self-reported by the nine-year old child, based on agreement with 8 items, for example “My parents understand me”, “I like my parents”, “My parents are usually unhappy or disappointed with what I do” (reverse-coded), “I get along well with my parents” (Cronbach alpha = 0.77). Parental knowledge of the child’s activities was self-reported by the 11 year-old child, using two items on whether parents knew the children they hung around with, and what they did with other children and interest in the child’s schooling (r = 0.29). Parental interest in the child’s schooling and religious observance were used as indicators of protective parental values. Parental interest in school was based on mother’s reports on six occasions from ages 6–11 (Cronbach alpha 0.81), while religious observance was based on mothers’ reports of how often the child attended a place of worship when the child was aged 9 and 11 years.

Turning to child characteristics, cognitive functioning was measured at age 8 using an abbreviated form of the Weschler Intelligence Scale for Children, WISC-IIIUK (Wechsler et al. 1992). Physical attractiveness was self-rated by the child at 9 years using agreement with 9 items, for example “I have a good looking body”, “I have nice features like nose, and eyes, and hair”, “I am ugly” (reversed), “Other kids think I am good looking”, Cronbach alpha = 0.87. Early romantic involvement was self-reported by children using at age 11 using an abbreviated form of the Adolescent Sexual Activity Index (Hansen et al. 1999), and was based on the number of behaviors from the following list: hugged, held hands, spent time alone with, kissed on the mouth, been kissed on the mouth and cuddled.

### Analysis

The first part of the analysis examined associations between different patterns of middle-childhood problems at each time point and early sexual behavior, using logistic regression. Next, associations between problem patterns and early sexual behavior were modeled after classifying each problem type as belonging to one of four patterns: none throughout, decreasing (present Time 1 only), increasing (present Time 2 only) and persistent (present Time 1 and Time 2). This analysis using problems defined using cut-offs was supplemented with analysis using continuous measures. Here, the effects of Time 1 problems together with change in problems from Time 1 to Time 2 were modeled. Lastly, multinomial regression explored associations between problems and adolescent risk subgroup membership. Analysis was conducted using Stata/IC version 12.1 (StataCorp, Texas). Two-tailed probabilities refer to Wald test statistics.

Levels of missing information were between 6 and 17 % for measures used. To decrease bias and increase statistical power, multiple imputation using the mi package in Stata 12.1 was used to impute missing values for the eligible data sample (White et al. 2011). Cases with missing outcome data (N = 59) were included in the imputation model, but excluded from analysis models to leave an analysis sample of 4,739 cases. The number of imputations was progressively increased to 100, when Monte-Carlo errors suggested reliable estimates. As univariate results using the imputed data set and complete cases were similar, we present models using the imputed data set only.

## Results

4,739 teenagers provided information on sexual behavior at the age 15 clinic. Almost a quarter (N = 1,141, 24 %) reported early sexual behavior, with 760 (16 %) reporting intercourse and oral sex, 91 (2 %) intercourse only and 290 (6 %) oral sex only. More girls than boys reported sexual behavior (26 % compared to 22 %, *p* < 0.001). Among the whole sample, 17 % had engaged at least one of four different risk behaviors at age 13 (smoking—8 %, drunk on alcohol—7 %, cannabis use—3 % or serious antisocial behavior—7 %), with 6 % engaging in two or more of these. Among teenagers reporting early sexual behavior, 33 % had engaged in any of these risk behaviors at age 13. The majority (88 %) of teenagers reporting early sexual behavior also reported at least one sexual risk, including 13 % using alcohol or drugs before sex, 36 % not using condom at most recent sex, 50 % not always using a condom, 38 % having more than one partner and 10 % engaging in sex by age 13. Four in ten teenagers who engaged in early sexual behavior reported three or more of these additional sexual risks (“high-risk sex”). Table 2 provides additional sample information, showing percentages engaging in early sexual behavior according to middle-childhood problems.

**Table 2:** Numbers in the analysis sample (N = 4,739) with middle-childhood problems, and percentages reporting early sexual behavior

Type of problem	Problem category	Time 1 (6–8 years)		Time 2 (10–11 years)	
		N (%) in problem category	% of problem category reporting early sexual behavior^a^	N in problem category	% of problem category reporting early sexual behavior^a^
Hyperactivity/inattention^b^	Normal	3,785 (91.1)	23.7	3,865 (94.6)	23.1
	Abnormal/borderline	371 (8.9)	22.0	220 (5.4)	24.3
Conduct problems^b^	Normal	3,250 (78.2)	22.5	3,524 (86.2)	22.0
	Abnormal/borderline	908 (21.8)	27.5	564 (13.8)	30.8
Peer relationship problems^b^	Normal	3,622 (87.2)	24.1	3,500 (85.5)	23.7
	Abnormal/borderline	533 (12.8)	19.9	592 (14.5)	19.9
Depressive symptoms^c^	Low	3,577 (87.5)	23.3	3,418 (84.1)	22.5
	High	512 (12.5)	23.6	646 (15.9)	26.5
School dislike^d^	Low	3,223 (71.1)	23.7	2,672 (64.1)	22.3
	High	1,308 (28.9)	24.0	1,498 (35.9)	26.0
School performance^d^	High	1,822 (45.5)	24.5	2,179 (49.6)	22.5
	Low	2,182 (54.5)	24.7	2,216 (50.4)	25.5

^a^Early sexual behavior denotes intercourse and/or oral sex in the last year, reported at age 15 clinic interview. Percentages exclude missing information

^b^Cut-offs for abnormal/borderline levels of conduct, hyperactivity/inattention and peer relationship problems were defined using recommended cut-offs for population norms (Goodman 1997)

^c^High levels of depressive symptoms were defined as top 10 % of scores, but percentages exceed 10 % due to tied scores

^d^Division into low and high was based on median scores

Associations between middle-childhood problems at each of the two time points (Time 1 6–8 years, Time 2 10–11 years) with early sexual behavior are shown in Table 3. Adjusted associations allow for other middle-childhood problems at the same time point, child’s gender, age in months and stage of puberty at the age 15 clinic assessment, measures of family adversity (mother’s education, maternal smoking, partner’s social class, biological father’s presence in household, household financial difficulties), parenting during middle-childhood (child’s relationship with parents, parental knowledge of the child’s activities, parental interest in child’s schooling, child’s attendance at place of worship) and child characteristics during middle-childhood (child’s early puberty, IQ, physical attractiveness, and early romantic behavior). Part A of Table 3 shows analyses using binary measures of problems. In the adjusted models, conduct problems were associated with an increased risk of early sexual behavior at both time points (odds ratio, OR, for Time 1 1.26, Time 2 1.37) while peer relationship problems were protective (OR 0.71 at both time points). At Time 2, school dislike was an additional predictor of early sexual behavior in adjusted analyses (OR 1.26). Part B of Table 3 shows analysis for problems measured with continuous scores, where odds ratios indicate the effect of a standard deviation increase in standardized score. This more sensitive analysis presents a similar picture to analysis using binary measures of problems, albeit with the addition of a protective effect of hyperactivity/inattention at Time 1 (OR 0.89, and a risk effect of depression at Time 2 (OR 1.10). The Time 2 effect of conduct problems reached only borderline (*p* = 0.06) statistical significance when using continuous scores.

**Table 3:** Associations between middle-childhood problems at each time point and early sexual behavior (imputed data set N = 4,739)

Time point	Problem (reference group)	Contrast	Unadjusted		Adjusted^a^	
			OR (95 % CI)	*p*	OR (95 % CI)	*p*
(A) *Analysis using cut-offs to measure problems*						
Time 1 (6–8 years)	Hyperactivity/inattention (normal)^b^	Borderline/abnormal	0.99 (0.82–1.21)	0.934	0.85 (0.68–1.52)	0.171
	Conduct problems (normal)^b^	Borderline/abnormal	1.30 (1.10–1.53)	0.002	1.26 (1.04–0.91)	0.018
	Peer problems (normal)^b^	Borderline/abnormal	0.78 (0.62–0.97)	0.027	0.71 (0.55–1.28)	0.006
	Depressive symptoms (low)^c^	High	1.02 (0.82–1.26)	0.879	1.01 (0.80–1.17)	0.930
	School dislike (low)^d^	High	1.01 (0.87–1.18)	0.854	0.99 (0.83–1.07)	0.873
	School performance (high)^d^	Low	1.04 (0.91–1.20)	0.550	0.89 (0.74–0.00)	0.212
Time 2 (10–11 years)	Hyperactivity/inattention (normal)^b^	Borderline/abnormal	1.15 (0.92–1.46)	0.226	0.82 (0.62–1.73)	0.174
	Conduct problems (normal)^b^	Borderline/abnormal	1.55 (1.28–1.88)	<0.001	1.38 (1.09–0.91)	0.007
	Peer problems (normal)^b^	Borderline/abnormal	0.82 (0.67–1.01)	0.068	0.71 (0.56–1.51)	0.007
	Depressive symptoms (low)^c^	High	1.30 (1.05–1.62)	0.017	1.16 (0.89–1.48)	0.282
	School dislike (low)^d^	High	1.24 (1.07–1.43)	0.004	1.26 (1.07–1.39)	0.006
	School performance (high)^d^	Low	1.20 (1.05–1.38)	0.009	1.15 (0.95–0.00)	0.145

Time point	Problem	Effect	Unadjusted		Adjusted^a^	
			OR (95 % CI)	*p*	OR (95 % CI)	*p*
(B) *Analysis using continuous scores to measure problems* ^e^						
Time 1 (6–8 years)	Hyperactivity/inattention	1 SD increase	0.99 (0.92–1.06)	0.721	0.89 (0.81–0.98)	0.013
	Conduct problems	1 SD increase	1.14 (1.07–1.22)	<0.001	1.17 (1.07–1.27)	<0.001
	Peer relationship problems	1 SD increase	0.94 (0.87–1.01)	0.089	0.89 (0.81–0.97)	0.007
	Depressive symptoms	1 SD increase	1.00 (0.93–1.08)	0.988	1.00 (0.92–1.08)	0.994
	School dislike	1 SD increase	1.02 (0.95–1.09)	0.664	1.00 (0.93–1.08)	0.930
	School performance	1 SD increase	0.99 (0.92–1.06)	0.729	1.09 (0.99–1.21)	0.092
Time 2 (10–11 years)	Hyperactivity/inattention	1 SD increase	1.09 (1.02–1.17)	0.012	0.98 (0.89–1.08)	0.667
	Conduct problems	1 SD increase	1.17 (1.09–1.25)	<0.001	1.09 (0.99–1.20)	0.064
	Peer relationship problems	1 SD increase	0.90 (0.83–0.97)	0.007	0.82 (0.75–0.90)	<0.001
	Depressive symptoms	1 SD increase	1.12 (1.05–1.20)	0.001	1.10 (1.00–1.20)	0.050
	School dislike	1 SD increase	1.10 (1.03–1.18)	0.008	1.11 (1.03–1.20)	0.010
	School performance	1 SD increase	0.95 (0.89–1.01)	0.126	1.00 (0.89–1.11)	0.954

^a^Adjusted for other problems at same time point, gender, age in months, stage of puberty at age 15 assessment, mother’s education, maternal smoking, partner’s social class, biological father’s presence in household, household financial difficulties, child’s relationship with parents, parental monitoring, maternal interest in child’s schooling, child’s attendance at place of worship, child’s early puberty, IQ, child’s physical attractiveness, and early romantic behavior

^b^Cut-offs for abnormal/borderline levels of conduct, hyperactivity/inattention and peer relationship problems were defined using recommended cut-offs for population norms (Goodman 1997)

^c^High levels of depressive symptoms were defined as top 10 % of scores, but percentages exceed 10 % due to tied scores

^d^Division into low and high was based on median scores

^e^Standardized scores were used to allow comparison of effects

Next, the onset and persistence of each problem was modeled by comparing three patterns (decreasing—Time 1 only, increasing—Time 2 only, and persistent—Time 1 and Time 2) to a reference group consisting of no problem at either time point, see Table 4 Part A. The adjusted model mutually adjusted for other middle-childhood problems and additional covariates, as for Table 3. In the adjusted model, only persistent conduct problems (8 % of the sample) had elevated odds of early sexual behavior (OR 1.75). Increasing school dislike (21 % of the sample), and worsening school performance (9 %) also increased risk of early sexual behavior (respective ORs 1.36 and 1.33). However, persistent hyperactivity/inattention and any pattern of peer problems reduced the likelihood of early sexual behavior.

**Table 4:** Associations between pattern of middle-childhood problems and early sexual behavior (imputed data set, N = 4,739)

Problem (reference group)	Contrast pattern (% with problem pattern^b^)	Unadjusted OR (95 % CI)	*p*	Adjusted^a^ OR (95 % CI)	*p*
(A) *Analysis using problems defined using cut-offs*					
Hyperactivity/inattention (none throughout)	Decreasing (9.9)	0.98 (0.76–1.25)	0.845	0.88 (0.67–1.17)	0.391
	Increasing (4.3)	1.28 (0.91–1.80)	0.149	0.96 (0.65–1.42)	0.850
	Persistent (5.9)	1.06 (0.78–1.44)	0.730	0.69 (0.48–1.00)	0.047
Conduct problems (none throughout)	Decreasing (13.8)	1.09 (0.88–1.35)	0.418	1.08 (0.85–1.37)	0.516
	Increasing (6.0)	1.31 (0.97–1.76)	0.079	1.16 (0.83–1.63)	0.383
	Persistent (8.2)	1.79 (1.41–2.28)	<0.001	1.75 (1.31–2.35)	<0.001
Peer relationship problems (none throughout)	Decreasing (7.8)	0.78 (0.58–1.03)	0.083	0.70 (0.51–0.96)	0.025
	Increasing (9.5)	0.84 (0.65–1.08)	0.178	0.74 (0.56–0.98)	0.037
	Persistent (5.3)	0.74 (0.53–1.05)	0.096	0.60 (0.40–0.89)	0.012
Depressive symptoms (low throughout)	Decreasing (9.8)	0.98 (0.76–1.26)	0.855	0.98 (0.75–1.29)	0.905
	Increasing (8.3)	1.30 (1.01–1.69)	0.043	1.16 (0.86–1.57)	0.336
	Persistent (2.9)	1.29 (0.85–1.94)	0.235	1.18 (0.73–1.90)	0.494
School dislike (low throughout)	Decreasing (13.7)	1.06 (0.85–1.32)	0.628	1.04 (0.82–1.32)	0.747
	Increasing (21.1)	1.32 (1.10–1.58)	0.003	1.36 (1.12–1.67)	0.002
	Persistent (15.1)	1.15 (0.94–1.41)	0.170	1.14 (0.91–1.44)	0.256
School performance (high throughout)	Improving (11.6)	0.99 (0.77–1.28)	0.965	0.86 (0.65–1.13)	0.282
	Worsening (9.5)	1.39 (1.08–1.79)	0.010	1.33 (1.00–1.76)	0.049
	Persistent (41.4)	1.16 (0.99–1.35)	0.065	1.03 (0.82–1.28)	0.826

Problem	Effect	Unadjusted		Adjusted^a^	
		OR (95 % CI)	*p*	OR (95 % CI)	*p*
(B) *Analysis using continuous problem scores* ^c^					
Hyperactivity Time 1	1 SD increase	0.90 (0.81–1.00)	0.061	1.11 (0.95–1.30)	0.169
Change in hyperactivity Time 1 to Time 2	1 SD increase	1.06 (0.94–1.18)	0.345	1.06 (0.92–1.23)	0.423
Conduct problems Time 1	1 SD increase	1.17 (1.05–1.31)	0.004	1.22 (1.06–1.40)	0.006
Change in conduct problems Time 1 to Time 2	1 SD increase	1.04 (0.94–1.15)	0.428	1.07 (0.94–1.21)	0.305
Peer problems Time 1	1 SD increase	0.85 (0.77–0.94)	0.001	0.78 (0.68–0.88)	<0.001
Change in peer problems Time 1 to Time 2	1 SD increase	0.88 (0.76–1.01)	0.076	0.92 (0.76–1.11)	0.391
Depression Time 1	1 SD increase	1.06 (0.95–1.19)	0.292	1.04 (0.94–1.15)	0.447
Change in depression Time 1 to Time 2	1 SD increase	1.08 (0.98–1.19)	0.101	1.07 (0.99–1.16)	0.110
School dislike Time 1	1 SD increase	1.08 (0.98–1.19)	0.117	1.08 (0.98–1.20)	0.106
Change school dislike Time 1 to Time 2	1 SD increase	1.11 (1.02–1.21)	0.016	1.12 (1.02–1.21)	0.012
School performance Time 1	1 SD increase	1.06 (0.94–1.19)	0.374	1.13 (0.99–1.28)	0.063
Change in school performance Time 1 to Time 2	1 SD increase	0.92 (0.80–1.04)	0.189	0.94 (0.83–1.08)	0.403

^a^Adjusted for other problems at same time point, gender, age in months, stage of puberty at age 15 assessment, mother’s education, maternal smoking, partner’s social class, biological father’s presence in household, household financial difficulties, child’s relationship with parents, parental monitoring, maternal interest in child’s schooling, child’s attendance at place of worship, child’s early puberty, IQ, child’s physical attractiveness, and early romantic behavior

^b^Problem patterns: decreasing (Time 1 only), increasing (Time 2 only), persistent (Time 1 and Time 2)

^c^All scores were standardized, to allow comparison of effects. Change scores were calculated by subtracting Time 1 from Time 2 scores, where positive scores indicate increases in problems but an improvement in school performance

Supplementary analyses were performed using continuous Time 1 scores and changes in scores from Time 1 to Time 2 (obtained by subtracting Time 1 from Time 2 scores, so that positive change scores denote increases in problems, although an improvement in school performance). Results are shown in Table 4 Part B. Note that the effect of Time 1 scores, when taking account of change in problems from Time 1 to Time 2, is similar to the concept of persistent problems defined using cut-off scores as in Table 4 Part A. The adjusted models produced similar findings to Table 3 for conduct problems and school dislike, namely Time 1 conduct problems and increasing school dislike from Time 1 to Time 2 each predicted early sexual behavior. A protective effect of Time 1 peer problems was found, though not of increasing peer problems. Effects of hyperactivity and school performance were not found for the models with continuous scores. Adding gender x problem interaction terms to the models in Tables 3 and 4 did not reveal any gender differences in the effect of middle-childhood problems on early sexual behavior (not shown).

Engaging in age 13 risk behavior was strongly predictive of early sexual behavior (OR 2.32, 95 % CI 1.79–3.01 adjusting for all covariates as for models in Tables 3 and 4), supporting the idea that this risk engagement forms a possible “pathway” to early sexual behavior. We next explored associations between problems and early sexual behavior, depending on whether teenagers reported other risk behavior in the early teens (=“high-risk pathway” to early sexual behavior). We divided teenagers into four subgroups: group 0 (67 % of the sample) with neither early sexual behavior nor age 13 risk; group 1 (early sexual behavior without age 13 risk, 17 %); group 2 (early sexual behavior and age 13 risk, 7 %); and group 3 (age 13 risk only, 9 %). Group 2 reported higher sexual risk-taking than group 1 (51 % of group 2 had a sexual risk score of 3 or more, compared to 34 % of group 1, *p* < 0.001). Multinomial regression compared predictors of risk subgroup membership, using group 0 as the reference. Table 5 presents relative risk ratios (RRR), mutually adjusting for middle-childhood problems and for all covariates as for Tables 3 and 4.

**Table 5:** Associations between patterns of middle-childhood problems and risk sub-group membership based on age 13 risk behavior: results of multinomial regression models (imputed data set, N = 4,739)

Reference group = no age 13 risk behavior^a^ or early sexual behavior		(1) Early sexual behavior without age 13 risk behavior		(2) Early sexual behavior with age 13 risk behavior		(3) Age 13 risk behavior only	
Problem (reference category)	Contrast pattern^b^	RRR (95 % CI)	*p*	RRR (95 % CI)	*p*	RRR (95 % CI)	*p*
Hyperactivity/inattention (none throughout)	Decreasing	0.87 (0.63–1.22)	0.428	0.89 (0.57–1.40)	0.622	0.98 (0.65–1.47)	0.917
	Increasing	0.96 (0.58–1.59)	0.874	1.22 (0.69–2.15)	0.502	1.57 (0.94–2.63)	0.082
	Persistent	0.63 (0.39–1.01)	0.057	0.90 (0.53–1.52)	0.697	1.28 (0.78–2.08)	0.327
Conduct problems (none throughout)	Decreasing	1.17 (0.88–1.54)	0.277	0.90 (0.60–1.34)	0.594	0.98 (0.69–1.40)	0.931
	Increasing	1.07 (0.70–1.63)	0.747	1.20 (0.72–2.02)	0.482	0.82 (0.48–1.39)	0.453
	Persistent	1.87 (1.31–2.67)	0.001	1.80 (1.12–2.91)	0.016	1.24 (0.79–1.94)	0.355
Peer relationship problems (none throughout)	Decreasing	0.70 (0.48–1.02)	0.066	0.73 (0.43–1.24)	0.246	1.15 (0.75–1.78)	0.522
	Increasing	0.71 (0.50–1.01)	0.059	0.78 (0.50–1.21)	0.260	1.02 (0.68–1.53)	0.925
	Persistent	0.64 (0.39–1.05)	0.080	0.57 (0.29–1.12)	0.104	1.17 (0.71–1.93)	0.534
Depressive symptoms (low throughout)	Decreasing	0.91 (0.65–1.26)	0.559	1.19 (0.78–1.82)	0.427	1.08 (0.72–1.64)	0.702
	Increasing	1.20 (0.83–1.72)	0.334	1.42 (0.88–2.28)	0.150	1.54 (1.02–2.34)	0.042
	Persistent	1.05 (0.58–1.92)	0.870	1.42 (0.70–2.88)	0.333	1.09 (0.55–2.19)	0.799
School dislike (low throughout)	Decreasing	1.06 (0.81–1.40)	0.668	1.23 (0.81–1.86)	0.326	1.56 (1.09–2.23)	0.016
	Increasing	1.33 (1.05–1.68)	0.019	1.98 (1.43–2.74)	<0.001	1.96 (1.44–2.67)	<0.001
	Persistent	1.10 (0.83–1.47)	0.509	1.89 (1.31–2.73)	0.001	2.42 (1.72–3.40)	<0.001
School performance (high throughout)	Improving	0.86 (0.62–1.18)	0.348	0.86 (0.54–1.37)	0.525	1.02 (0.67–1.58)	0.913
	Worsening	1.25 (0.90–1.74)	0.178	1.50 (0.95–2.38)	0.080	1.00 (0.63–1.61)	0.988
	Persistent	0.99 (0.77–1.29)	0.969	1.12 (0.77–1.62)	0.552	1.06 (0.75–1.49)	0.752

RRR denotes relative risk ratio. Models adjusted for other problems at same time point, gender, age in months, stage of puberty at age 15 assessment, mother’s education, maternal smoking, partner’s social class, biological father’s presence in household, household financial difficulties, child’s relationship with parents, parental monitoring, maternal interest in child’s schooling, child’s attendance at place of worship, child’s early puberty, IQ, child’s physical attractiveness, and early romantic behavior. ^a ^Risk behavior denotes one or more of smoking, drinking, cannabis use or serious antisocial behaviour (see text for full definition). ^b^ Problem patterns: decreasing (Time 1 only), increasing (Time 2 only), persistent (Time 1 and Time 2)

Persistent early conduct problems and increasing school dislike predicted membership of both subgroups reporting early sexual behavior (group 1 RRR persistent conduct problems 1.87, *p* < 0.001 and increased school dislike 1.33, *p* < 0.05; group 2 corresponding RRRs were 1.80 *p* < 0.05 and 1.98 *p* < 0.001). Group 2, the higher risk group engaging in prior risk behavior at age 13, was further characterized by an effect of persistent early school dislike (RRR 1.89 *p* < 0.001). Comparing groups 1 and 2 by resetting the reference indicated that effects of increased and persistent early school dislike were stronger for group 2 (differences *p* < 0.05). Inspection of covariates found that early romantic behavior, father absence and lower parental monitoring had stronger effects in group 2 than group 1 (not shown).

Persistent early school dislike also was found for group 3 (age 13 risk without early sexual behavior). However, this group did not have persistent conduct problems (difference between group 3 and combined groups 1 and 2 bordered significance, *p* = 0.09). Comparing groups 3 and 2 found no differences in middle-childhood problems, although inspection of covariates found that effects of age, early romantic behavior and puberty, greater physical attractiveness, lower parental monitoring and less supportive parent–child relationship were stronger in group 2 (not shown). Additional sensitivity analyses conducted with alternative risk group formulations, based on early sexual behavior and using each of the individual age 13 risk behaviors in the composite measure in turn to define the “high-risk” pathway, produced similar results (not shown).

An alternative formulation of subgroups 1 and 2, based on their sexual risk-taking, gave similar results (see Table 6 for relative risk ratios, adjusted as for Table 5). Group 2 (with additional higher-risk sexual behavior) membership was predicted by persistent early conduct problems (RRR 2.23, *p* < 0.05), persistent early school dislike (RRR 1.55, *p* < 0.01) and increasing school dislike (RRR 1.45, *p* < 0.05). Only increasing school dislike was clearly associated with group 1 (with lower additional sexual risk behavior) membership (RRR 1.49, *p* < 0.001) as here the effect of persistent early conduct problems reached only borderline statistical significance (*p* = 0.08).

**Table 6:** Associations between patterns of middle-childhood problems and risk sub-group membership based on sexual risk-taking: results of multinomial regression (imputed data set, N = 4,739)

Reference group = no age 13 risk behavior or early sexual behavior		(1) Early sexual behavior with lower sexual risk-taking^a^		(2) Early sexual behavior with higher sexual risk-taking		(3) Age 13 risk behavior only^b^	
Problem	Contrast pattern^c^	RRR (95 % CI)	*p*	RRR (95 % CI)	*p*	RRR (95 % CI)	*p*
Hyperactivity/inattention (none throughout)	Decreasing	0.98 (0.66–1.45)	0.916	1.00 (0.62–1.61)	0.994	0.95 (0.57–1.59)	0.840
	Increasing	0.75 (0.54–1.04)	0.088	0.88 (0.60–1.28)	0.499	1.18 (0.81–1.72)	0.388
	Persistent	0.53 (0.08–3.66)	0.519	2.71 (0.82–9.00)	0.103	2.32 (0.70–7.73)	0.170
Conduct problems (none throughout)	Decreasing	0.89 (0.64–1.23)	0.481	1.37 (0.96–1.95)	0.081	1.14 (0.80–1.63)	0.473
	Increasing	1.18 (0.88–1.59)	0.258	1.36 (0.95–1.94)	0.092	0.89 (0.62–1.28)	0.522
	Persistent	1.76 (0.93–3.31)	0.080	2.23 (1.08–4.58)	0.029	1.40 (0.61–3.22)	0.430
Peer relationship problems (none throughout)	Decreasing	0.61 (0.38–0.97)	0.035	0.89 (0.55–1.42)	0.623	1.16 (0.74–1.83)	0.522
	Increasing	0.78 (0.56–1.08)	0.134	0.73 (0.49–1.09)	0.123	0.99 (0.68–1.46)	0.979
	Persistent	0.80 (0.26–2.43)	0.694	0.89 (0.25–3.12)	0.859	1.23 (0.41–3.70)	0.718
Depressive symptoms (low throughout)	Decreasing	1.01 (0.73–1.39)	0.946	0.95 (0.64–1.41)	0.786	1.06 (0.70–1.60)	0.774
	Increasing	1.21 (0.85–1.73)	0.291	1.37 (0.91–2.06)	0.130	1.65 (1.11–2.45)	0.013
	Persistent	1.29 (0.74–2.25)	0.378	1.12 (0.56–2.21)	0.751	1.26 (0.64–2.49)	0.499
School dislike (low throughout)	Decreasing	1.14 (0.87–1.51)	0.345	1.04 (0.73–1.49)	0.825	1.58 (1.11–2.24)	0.011
	Increasing	1.49 (1.17–1.89)	0.001	1.45 (1.08–1.95)	0.015	1.95 (1.41–2.69)	<0.001
	Persistent	1.19 (0.89–1.60)	0.236	1.55 (1.11–2.15)	0.010	2.41 (1.72–3.37)	<0.001
School performance (high throughout)	Improving	0.86 (0.61–1.20)	0.374	0.89 (0.60–1.32)	0.550	1.04 (0.69–1.56)	0.850
	Worsening	1.18 (0.84–1.66)	0.342	1.43 (0.96–2.12)	0.079	1.03 (0.64–1.68)	0.893
	Persistent low	1.02 (0.78–1.32)	0.897	0.97 (0.70–1.35)	0.871	1.10 (0.78–1.53)	0.590

RRR = relative risk ratio. Adjusted for other problems at same time point, gender, age in months, stage of puberty at age 15 assessment, mother’s education, maternal smoking, partner’s social class, biological father’s presence in household, household financial difficulties, child’s relationship with parents, parental monitoring, maternal interest in child’s schooling, child’s attendance at place of worship, child’s early puberty, IQ, child’s physical attractiveness, and early romantic behavior

^a^Sexual risk based on very early sexual behavior (age 13), condom use, use of alcohol/drugs at sex, number of partners. See text for full definition

^b^Group 3 defined as for Table 5

^c^Problem patterns: decreasing (Time 1 only), increasing (Time 2 only), persistent (Time 1 and Time 2)

Supplementary analyses conducted using continuous problem scores and changes in these scores are provided in Online Resource 2. Results were similar to the analyses reported in Tables 5 and 6. In particular, T1 school dislike and increasing school dislike were associated with both higher-risk subgroups amongst teenagers reporting early sexual behavior. Time 1 conduct problems appeared associated with early sexual behavior regardless of prior risk involvement, although the coefficient for the higher-risk subgroup was not statistically significant and there was an additional risk of increased hyperactivity/inattention for this subgroup. Additionally, the lower-risk subgroup without prior age 13 risk was characterized by an increased risk of depression. As before, Time 1 conduct problems were associated with increased sexual risk-taking, but not clearly with lower sexual risk-taking.

## Discussion

This study investigated associations between several types of middle-childhood problem and early sexual behavior, extending information from previous studies with a narrower focus on externalizing problems, and allowing for a greater range of confounding influences. Conduct problems and disliking primary school were both independent predictors of early sexual behavior, adding to existing findings on middle childhood conduct problems (Dupéré et al. 2008; McLeod and Knight 2010; Ramrakha et al. 2007; Schofield et al. 2008b), and establishing school dislike as an earlier risk factor than previously known. The results lend support to the idea that more than one type of middle-childhood problem may increase the risk of early adolescent sexual behavior, even after allowing for many shared influences on problems and early sexual behavior such as family deprivation, parenting and other child characteristics including early puberty. For conduct problems, our study builds on existing research by indicating the particular importance of problem onset and persistence. Other studies have not examined different trajectories of conduct problems in relationship to the initiation of early sexual activity. The influence of conduct problems on early sexual behavior in our population study could be traced to the early primary school years, in line with a high-risk sample (Schofield et al. 2008a). However, we found that there was a risk of early sexual behavior only when early conduct problems persisted throughout primary school, and not for problems with a later onset or for problems limited to early primary school. This is in line with research suggesting lesser or negligible impacts of both child-limited and later onset conduct problems on young adult health risks (Odgers et al. 2008). The effect size that we found for persistent conduct problems was below that found for conduct problems measured across middle childhood in two other population studies (Ramrakha et al. 2007; Dupéré et al. 2008), but similar to that found for conduct problems at age 10–11 years in another study adjusting for a wider range of externalizing and internalizing problems (McLeod and Knight 2010). In line with existing studies, we found no gender differences in the effect of conduct problems on early sexual behavior, even though there were gender differences in the prevalence of both early conduct problems and sexual behavior.

In line with research on the development of antisocial behavior (Laird et al. 2001), later-onset problems with school engagement added to risk from early conduct problems. School dislike was not generally important as a predictor of early sexual behavior until 10–11 years. This is still earlier than previously established associations between poor school engagement in early adolescence and sexual behavior, for example Bonell et al. 2005, and supports the notion that middle-childhood problems may accumulate risk. This might happen in two ways. Children with early conduct problems may experience later difficulties fitting in at school, which may add to the risk posed by conduct problems. Additionally, school dislike in late primary may identify further children, without conduct problems, who are nevertheless at risk of early sexual behavior. The effect of disliking school on early sexual behavior was found even though we allowed for several other confounding influences, including parental interest in school (Fredricks et al. 2004), parent–child relationship quality (Dotterer 2008), conduct problems (Li 2011) and peer relationship problems (Betts et al. 2012). There were no gender differences in the effect of disliking primary school on early sexual behavior, even though some adolescent studies have found effects for girls only (e.g. Whitbeck et al. 1999).

There are several possible mechanisms for associations between disliking primary school and adolescent risk behaviors, such as early sexual activity. First, there may be repercussions for school behavioral engagement and performance (Ladd and Dinella 2009), as children who are demotivated apply themselves less to their school work, and their school grades suffer. In turn, poor performance may reduce academic expectations and aspirations, and facilitate adoption of less conventional norms leading to risk behavior. Poor performance also might lead to risk behavior via depression, induced by negative self-affect and loss of perceived control (Moilanen et al. 2010). Reduced performance may not in itself carry much risk until secondary school: our study did not find that lower school performance in primary school age children was associated with early sexual behavior. This is in contrast to findings for a role for low school performance in the early teens (Laflin et al. 2008), and could reflect an underlying trend for school engagement to drive achievement, rather than the reverse (Valiente et al. 2007). Second, late primary school is likely to mark the start of pubertal development, greater independence from parents and increased peer influence. A child who does not enjoy late primary school may find it increasingly attractive and possible to gravitate towards the company of other like-minded, delinquent peers. In the early teens, lower school engagement is associated with higher rates of delinquency and drug use (Li et al. 2011), which, in turn, could lead to early sexual behavior. Importantly in this United Kingdom study, school dislike was found to be a risk factor before children moved from primary to secondary school. Particular difficulties over the transition to secondary school may represent a third way in which dislike during late primary school is translated into adolescent risk behavior. At transition, the child (typically) leaves a relatively small primary class, taught for much of the day by the same teacher, for a school several times bigger with different teachers for different subjects addressing some new or unfamiliar topic areas. Disliking the later stages of primary school may inhibit successful transition (West et al. 2010), as the child who dislikes school may find social integration into dominant “healthy” peer groups more difficult, in addition to experiencing more challenges from the different learning environment.

Similar predictors of high-risk subgroup membership were found using our two alternative risk group classifications, lending support to the idea that adolescents following a high risk pathway to early sexual behavior via other risk behavior in their early teens are also those exposed to greater sexual risk-taking. Persistent early conduct problems and two patterns of school dislike (persistent early, and increasing) appeared to have distinct, although overlapping, associations with different teenage risk subgroups. Conduct problems had a more specific association with early sexual behavior than with other types of adolescent involvement in risk, a finding that lends support to speculation that there may be shared temperamental attributes underlying both behaviors, perhaps related to poor behavioral control, stress-reduction or sensation-seeking (Timmermans et al. 2008). School dislike in late primary school was universally associated with risk, although more strongly with higher-risk subgroups. School dislike may be more allied with the development of unconventional norms than conduct problems. Of particular interest in our study is the finding that persistent school dislike emerging in early primary years was associated with higher-risk subgroups. Little is currently known about trajectories of school engagement in childhood (Fredricks et al. 2004), but our findings seem to tally with recent work on adolescent trajectories suggesting that decreasing emotional engagement is predictive of highest rates of risk behavior and depression (Li and Lerner 2011). It seems likely that early, sustained school dislike may be a marker for even greater alienation from conventional institutions than dislike arising later in primary school. Persistent early school dislike signaled substance use or antisocial behavior in the early teens; whether this led to early sexual behavior seemed to depend on the presence of other biological and social factors, such as early puberty, physical attractiveness, low parental monitoring and early dating, as well as on whether conduct problems were present. A useful extension of the current study, which is concerned with risk posed by each problem type separately, would be to examine how different clusters of middle-childhood problems, together with other important biological and social factors, predict early sexual activity. Our overall finding, that a greater number of early and sustained problems predicted higher risk subgroups within the group of adolescents reporting early sexual behavior, is consistent with a greater accumulation of childhood risk factors for higher-risk, life-course persistent antisocial behavior (Moffitt and Caspi 2001).

Our study did not find clear associations between other types of middle-childhood problems and increased risk of early sexual behavior. In agreement with some (Ramrakha et al. 2007) but not all (Schofield et al. 2008a; Galéra et al. 2010; McLeod and Knight 2010) previous research, we did not find a consistent role for middle childhood hyperactivity/inattention. Other work looking at associations between middle-childhood problems and a range of adult outcomes, controlling for many other family and child characteristics, found effects of attentional problems were confined to educational and employment outcomes, rather than risk behaviors including sexual health (Fergusson et al. 2005). Differences between our study and those with positive findings might reflect our use of the Strengths and Difficulties questionnaire, or the wider range of problems studied here, including school problems. Information on associations between depression and early sexual initiation is limited to a few studies of early teens (Zimmer-Gembeck and Helfand 2008), with girls appearing most vulnerable. However, we did not find a consistent independent role for depression in younger age groups of either gender. Lastly, although a study of 10–11 year olds found no effect of peer problems (McLeod and Knight 2010), some research on the development of adolescent risk has stressed the role of peer rejection in the formation of ties to delinquent peers (Deater-Deckard 2001). In contrast, our study found that peer relationship problems at any time in primary school were protective. It suggests that children who find it difficult to make friends or be accepted by their peers are less likely to engage in early sexual activity, regardless of other problems. This could indicate the importance of sociability and popularity for romantic (Connolly et al. 2000) and sexual behavior (Meschke et al. 2000). It also could suggest an inhibitory effect of shy temperament (Galéra et al. 2010).

### Limitations and Future Directions

This study has several limitations related to measurement issues and sampling. The study relied on self-reported measures of sexual and other risk behaviors, and some attitudinal measures (including school dislike) not validated elsewhere. The outcome measure of sexual behavior in the last year referred only to sexual behavior with other young people. It will therefore have omitted sexual relations with adults, including potentially more coercive and abusive relationships. Although very early sexual behavior was measured at the age 13 clinic, it is possible that the study omitted some teenagers who experienced sex after the age 13 clinic, but who did not repeat the experience less than a year before the age 15 clinic. Mothers’ reports of children’s behavioral and emotional problems are subject to bias. For these problems, but not for child-reported school dislike, the use of child-reported outcomes helped to avoid difficulties associated with the same observer reporting both predictors and outcomes. For some measures, including depression, school performance and school dislike, use of different scales at the two time points means that change estimates are subject to error. Findings also may to some extent reflect cut-offs used, although analyses using continuous scores for problems substantiated results using binary measures of problems. For sexual risk subgroups, alternative risk group classifications gave similar findings. The eligible sample contained fewer teenagers from less affluent social groups and fewer with childhood problems than the full data set, potentially restricting generalizability of results; although within our sample, bias and loss of power due to missing information was addressed through multiple imputation. Even so, lack of power may have restricted the ability to detect gender differences. Substantial strengths include the longitudinal design, use of linked data for school attainment, and adjustment for many confounders. Residual confounding remains a possibility. For example, there may be early familial factors (Donahue et al. 2013) or temperamental attributes (Stringaris et al. 2010) underlying conduct problems and sexual behavior, and emotional disengagement with school may be reflected more generally in lower competence and control, or poorer relationships with others (Fredricks et al. 2004).

More research is required using a longer follower-up into late adolescence, in order to confirm whether the middle-childhood problems studied here do clearly differentiate early from more normative sexual timing, as suggested by a recent review (Zimmer-Gembeck and Helfand 2008). Here, we were only able to examine predictors of early timing but, if the review is correct, middle-childhood problems would not be associated with more normative timing, between 16 and 18 years. Research is also required to unravel the mechanisms by which middle-childhood problems may impact on early sexual behavior via processes in early adolescence. For conduct problems, some research has begun to explore how these may lead to sexual behavior via social processes. One general population study found that associations between conduct problems and early sexual behavior could partly be explained by closer ties with delinquent peer groups and poorer relationship with parents in the early teens (Ramrakha et al. 2007). There may be a role for the neighborhood environment, from research showing neighborhood poverty accelerates early sexual behavior through affiliations with delinquent peers (Dupéré et al. 2008). Another study found a role for school maladjustment in linking conduct problems and early sexual behavior (Schofield et al. 2008a). It is also possible that conduct problems lead to early sexual behavior via depression, and further testing of psychosocial deficits models for the direct effects of both conduct problems and depression on early sexual behavior seem desirable, particularly since poor self-regulation seems to be strongly predictive of greater sexual risk-taking (Raffaelli and Crockett 2003). Behavioral impulsivity seems to be a dominant characteristic of children with persistent conduct problems (Ackerman et al. 2003), and this characteristic has been linked to early sexual behavior (Khurana et al. 2012). Research is needed to understand processes linked to early school dislike, as this appears to capture a group of children at risk for early sexual behavior who are not necessarily characterized by early behavioral or peer relationship problems. Emotional engagement with school is not currently well-defined or understood (Fredricks et al. 2004; Li 2011), and the global measure used here might reflect disaffection with various aspects of school life, including classmates, teachers and academic work.

In conclusion, this study suggests several directions for further research to explore the origins and consequences of both conduct problems and school dislike, in order to understand mechanisms for associations with early sexual behavior. It adds to concerns raised over links between middle-childhood problems and other teenage risk behavior, such as delinquency (Broidy et al. 2003) and drinking (Macleod et al. 2008), while suggesting that early sexual behavior may differ from other risk behavior in the protective effect of peer relationship problems. The findings also point to opportunities for identification and targeted intervention to avoid early sexual activity and associated risk-taking. In recent years, there has been a growing recognition of the value of interventions to reduce childhood conduct problems, with recent UK guidelines on the recognition, intervention and management of children with conduct problems covering both school and parent-focused programs (Pilling et al. 2013). This study supports the idea that targeting children with early, persistent conduct problems may be particularly valuable. Identifying poor emotional engagement with primary school may present some challenges, particularly in young children, or if dislike of school does not clearly translate to reduced behavioral engagement. The study suggests a need to distinguish disliking school from other problems the child may have, particularly with peer relationships. In targeting school engagement, it may be possible to build on existing work on enhancing school readiness through building socio-emotional competence in young children (Sheridan et al. 2010). Overall, our study reinforces the notion that multifaceted, early approaches involving primary schools, parents and children themselves may be optimal. These could target both early conduct problems and low emotional engagement with school to prevent problems becoming ingrained, with an additional focus on children who become disaffected with school just before puberty.

## Electronic supplementary material

Below is the link to the electronic supplementary material.

Supplementary material 1 (DOCX 14 kb)

Supplementary material 2 (DOCX 19 kb)

## Footnotes

1 For further details see https://www.gov.uk/national-curriculum/overview
