# Supplementary material for: Which Behavioral, Emotional and School Problems in Middle-Childhood Predict Early Sexual Behavior?
Source: J Youth Adolesc. 2013 Jul 4;43(4):507–27. doi: 10.1007/s10964-013-9973-x (PMC3949009; doi:10.1007/s10964-013-9973-x)
Supplement: Supplementary file 1 — Supplementary material 1 (DOCX 14 kb) [file 10964_2013_9973_MOESM1_ESM.docx]

| **Online Resource 1**  ***Sample Information for Covariates*** |  |
| --- | --- |
| **Measure (range)** | **Mean (SD) or N, % in sample with characteristic** |
| Gender | N=2552, 53% |
| Age in months at age 15 clinic (180-205 months) | 185.36 (3.58) |
| Early pubertal development (Tanner^a^ stage of development 1-5) | 2.19 (0.99) |
| Pubertal development at age 15 clinic (Tanner stage of development 1-5) | 4.29 (0.65) |
| Physical attractiveness (1,low -5,high) | 3.82 (0.77) |
| Early romantic behaviour (range 0-6) | 1.08 (1.84) |
| Religious observance  (3 point scale from 1="often" to 3="never"). | 2.35 (0.72) |
| IQ | 107.00 (15.93) |
| Mother's education level ( degree vs. below degree) | 3805, 82% below degree |
| Father's social class (non-manual vs. manual) | 1535, 36% manual |
| Mother ever smoked  (no/yes) | 1940, 41% ever smoked |
| Father resident in household  (present/absent) | 986, 21% absent |
| Financial difficulties  (0-15) | 2.58 (2.82) |
| Child's relationship with parents (1, poor to 5, good) | 4.66 (0.47) |
| Parental knowledge of child's activities  (range 1, low to 4, high) | 3.39 (0.54) |
| Parental interest in school  (range 1, low to 3, high) | 1.09 (0.22) |
