# Supplementary material for: Which Behavioral, Emotional and School Problems in Middle-Childhood Predict Early Sexual Behavior?
Source: J Youth Adolesc. 2013 Jul 4;43(4):507–27. doi: 10.1007/s10964-013-9973-x (PMC3949009; doi:10.1007/s10964-013-9973-x)
Supplement: Supplementary file 2 — Supplementary material 2 (DOCX 19 kb) [file 10964_2013_9973_MOESM2_ESM.docx]

| **Online Resource 2** |  |  |  |  |  |  |  |
| --- | --- | --- | --- | --- | --- | --- | --- |
| ***Risk Sub-group Models using Continuous Problem Scores, Imputed data set N=4739*** | | | | | | | |
| **A) Risk groups according to Early Sexual Behavior and Age 13 Risk Behavior** | |  |  |  |  |  |  |
| **Reference group=No Age 13 Risk Behavior^1^ or Early Sexual Behavior** | | **1) Early Sexual Behavior without Age 13 Risk Behavior** | | **2) Early Sexual Behavior with Age 13 Risk Behavior** | | **3) Age 13 Risk Behavior Only** | |
| **Problem** | **Effect** | **RRR (95% CI)** | ***p*** | **RRR (95% CI)** | ***p*** | **RRR (95% CI)** | ***p*** |
| Hyperactivity Time 1 | 1 SD increase | 0.88 (0.77 - 1.00) | 0.053 | 1.00 (0.83 - 1.19) | 0.980 | 1.06 (0.90 - 1.25) | 0.474 |
| Change in hyperactivity Time 1 to Time 2 | 1 SD increase | 1.01 (0.87 - 1.16) | 0.921 | 1.26 (1.05 - 1.50) | 0.011 | 1.14 (0.96 - 1.35) | 0.144 |
| Conduct problems Time 1 | 1 SD increase | 1.21 (1.06 - 1.37) | 0.005 | 1.16 (0.97 - 1.38) | 0.107 | 1.04 (0.88 - 1.22) | 0.653 |
| Change in conduct problems Time 1 to Time 2 | 1 SD increase | 1.05 (0.93 - 1.18) | 0.455 | 1.12 (0.95 - 1.33) | 0.185 | 1.10 (0.94 - 1.28) | 0.225 |
| Peer problems Time 1 | 1 SD increase | 0.79 (0.69 - 0.91) | 0.001 | 0.78 (0.65 - 0.94) | 0.009 | 1.01 (0.87 - 1.18) | 0.890 |
| Change in peer problems Time 1 to Time 2 | 1 SD increase | 0.84 (0.74 - 0.94) | 0.004 | 0.87 (0.73 - 1.02) | 0.087 | 0.99 (0.87 - 1.13) | 0.922 |
| Depression Time 1 | 1 SD increase | 1.08 (0.93 - 1.24) | 0.309 | 1.17 (0.97 - 1.40) | 0.094 | 1.14 (0.97 - 1.34) | 0.113 |
| Change in depression Time 1 to Time 2 | 1 SD increase | 1.13 (1.00 - 1.27) | 0.042 | 1.10 (0.94 - 1.29) | 0.220 | 1.10 (0.96 - 1.26) | 0.156 |
| School dislike Time 1 | 1 SD increase | 1.06 (0.94 - 1.19) | 0.375 | 1.43 (1.21 - 1.69) | <0.001 | 1.58 (1.36 - 1.84) | <0.001 |
| Change school dislike Time 1 to Time 2 | 1 SD increase | 1.09 (0.98 - 1.20) | 0.116 | 1.33 (1.16 - 1.53) | <0.001 | 1.31 (1.16 - 1.49) | <0.001 |
| School performance Time 1 | 1 SD increase | 1.05 (0.91 - 1.21) | 0.519 | 1.01 (0.82 - 1.23) | 0.940 | 0.92 (0.76 - 1.11) | 0.396 |
| Change in school performance Time 1 to Time 2 | 1 SD increase | 0.91 (0.78 - 1.07) | 0.257 | 0.88 (0.71 - 1.09) | 0.236 | 0.95 (0.77 - 1.16) | 0.608 |
|  |  |  |  |  |  |  |  |
| **B) Alternative Risk group Classification of groups 1 and 2 according to Sexual Risk-taking** | | |  |  |  |  |  |
| **Reference group= No Age 13 Risk Behavior or Early Sexual Behavior** | | **1) Early Sexual Behavior with Lower Sexual Risk-taking^2^** | | **2) Early Sexual Behavior with Higher Sexual Risk-taking** | | **3) Age 13 Risk Behavior Only ^3^** | |
| **Problem** | **Effect** | **RRR (95% CI)** | ***p*** | **RRR (95% CI)** | ***p*** | **RRR (95% CI)** | ***p*** |
| Hyperactivity Time 1 | 1 SD increase | 0.95 (0.80 - 1.13) | 0.547 | 0.87 (0.70 - 1.09) | 0.222 | 1.06 (0.85 - 1.33) | 0.578 |
| Change in hyperactivity Time 1 to Time 2 | 1 SD increase | 1.11 (0.94 - 1.33) | 0.223 | 1.02 (0.82 - 1.28) | 0.847 | 1.10 (0.88 - 1.37) | 0.409 |
| Conduct problems Time 1 | 1 SD increase | 1.05 (0.89 - 1.25) | 0.553 | 1.35 (1.10 - 1.66) | 0.004 | 1.02 (0.83 - 1.26) | 0.840 |
| Change in conduct problems Time 1 to Time 2 | 1 SD increase | 0.96 (0.82 - 1.13) | 0.637 | 1.18 (0.98 - 1.43) | 0.084 | 1.04 (0.85 - 1.27) | 0.719 |
| Peer problems Time 1 | 1 SD increase | 0.77 (0.64 - 0.92) | 0.004 | 0.81 (0.65 - 1.02) | 0.068 | 1.05 (0.86 - 1.28) | 0.633 |
| Change in peer problems Time 1 to Time 2 | 1 SD increase | 0.86 (0.74 - 1.00) | 0.056 | 0.87 (0.71 - 1.06) | 0.154 | 1.00 (0.83 - 1.19) | 0.967 |
| Depression Time 1 | 1 SD increase | 1.11 (0.94 - 1.33) | 0.219 | 1.09 (0.88 - 1.36) | 0.436 | 1.12 (0.91 - 1.38) | 0.270 |
| Change in depression Time 1 to Time 2 | 1 SD increase | 1.09 (0.94 - 1.25) | 0.255 | 1.05 (0.88 - 1.26) | 0.606 | 1.07 (0.90 - 1.27) | 0.450 |
| School dislike Time 1 | 1 SD increase | 1.15 (0.98 - 1.35) | 0.095 | 1.24 (1.01 - 1.51) | 0.040 | 1.65 (1.36 - 2.01) | <0.001 |
| Change school dislike Time 1 to Time 2 | 1 SD increase | 1.10 (0.96 - 1.25) | 0.168 | 1.22 (1.04 - 1.44) | 0.017 | 1.31 (1.11 - 1.54) | 0.001 |
| School performance Time 1 | 1 SD increase | 1.06 (0.88 - 1.29) | 0.528 | 1.33 (1.04 - 1.72) | 0.025 | 0.89 (0.70 - 1.14) | 0.355 |
| Change in school performance Time 1 to Time 2 | 1 SD increase | 0.89 (0.72 - 1.08) | 0.236 | 0.99 (0.76 - 1.30) | 0.947 | 0.90 (0.70 - 1.16) | 0.415 |

Note: RRR denotes relative risk ratio, SD denotes standard deviation. All problem scores were standardised, to allow comparison of effects. Change scores were calculated by subtracting Time 1from Time 2 scores, where positive scores indicate increases in problems but an improvement in school performance. Models adjusted for other problems at same time point, gender, age in months, stage of puberty at age 15 clinic assessment, mother's education, maternal smoking, partner's social class, biological father's presence in household, household financial difficulties, child's relationship with parents, parental monitoring, maternal interest in child's schooling, child's attendance at place of worship, child's early puberty, IQ, child's physical attractiveness, and early romantic behaviour. ^1^Risk behaviour denotes one or more of smoking, drinking, cannabis use or serious antisocial behaviour (see text for full definition). ^2^ Problem patterns: decreasing (Time 1 only), increasing (Time 2 only), persistent (Time 1 and Time 2). ^1^ Sexual risk based on very early sexual behavior (age 13), condom use, use of alcohol/drugs at sex, number of partners. See text for full definition. ^3^ Group 3 defined as for Part A.
